# A Systematic Review of Rhubarb (a Traditional Chinese Medicine) Used for the Treatment of Experimental Sepsis

**DOI:** 10.1155/2015/131283

**Published:** 2015-08-03

**Authors:** Fang Lai, Yan Zhang, Dong-ping Xie, Shu-tao Mai, Yan-na Weng, Jiong-dong Du, Guang-ping Wu, Jing-xia Zheng, Yun Han

**Affiliations:** Department of Intensive Care Unit, Guangdong Provincial Hospital of Chinese Medicine, Guangzhou 510145, China

## Abstract

Sepsis is a global major health problem in great need for more effective therapy. For thousands of years, Rhubarb had been used for various diseases including severe infection. Pharmacological studies and trials reported that Rhubarb may be effective in treating sepsis, but the efficacy and the quality of evidence remain unclear since there is no systematic review on Rhubarb for sepsis. The present study is the first systematic review of Rhubarb used for the treatment of experimental sepsis in both English and Chinese literatures by identifying 27 studies from 7 databases. It showed that Rhubarb might be effective in reducing injuries in gastrointestinal tract, lung, and liver induced by sepsis, and its potential mechanisms might include reducing oxidative stress and inflammation, ameliorating microcirculatory disturbance, and maintaining immune balance. Yet the positive findings should be interpreted with caution due to poor methodological quality. In a word, Rhubarb might be a promising candidate that is worth further clinical and experimental trials for sepsis therapy.

## 1. Introduction

Sepsis is a clinical syndrome resulting from an inflammatory response to a severe infection. It is characterized by systemic manifestations of infection, including vasodilation, leukocyte accumulation, and increased microvascular permeability in tissues remote from the infection. Nowadays, it is a global major health problem with great incidence rates, high mortality rates, and huge cost consumption. Although the guidelines of “Surviving Sepsis Campaign” have led to advances in sepsis therapy and thus a reduction in sepsis mortality [1], the mortality rates are still high, and there is an absolute necessity for more effective therapy [2].

In the course of human history, infectious diseases have always been a major subject, and sepsis was a whole new concept extracted from that in the 1990s, but it was never a new thing to human beings as to the disease itself [2] and it must be even more common in the ancient times due to poor environmental sanitation and lack of modern medical technology. Since ancient times, great efforts have been made to struggle with sepsis in China. The oldest and greatest existing Chinese medical classic,* Huangdi's Internal Classic* (*Huang Di Nei Jing*), first recorded different sepsis-related symptoms.* Treatise on Febrile Diseases* (*Shan Han Lun*), written in the Eastern-Han dynasty, is the oldest existing Chinese medical monograph concerning infectious disease. In the Jin and Yuan dynasties, seasonal febrile disease theory formed and developed, further improving the Chinese clinical science of febrile diseases.

Rhubarb (*Da Huang*), one of the most popular traditional Chinese medicines used to control various diseases for thousands of years, is made of roots and rhizomes of* Rheum palmatum *L.,* Rheum tanguticum *Maxim. ex Balf.,* Rheum undulatum*, or* Rheum officinale *Baill. [3, 4]. It was first documented in* Shen Nong's Herbal Classic* (*Shen Nong Ben Cao Jing*), the oldest Chinese materia medica, and later in* Huangdi's Internal Classic* (*Huang Di Nei Jing*) and was commonly employed in* Treatise on Febrile Diseases* (*Shan Han Lun*) and seasonal febrile disease theory as a purgative and bactericidal agent to reduce fever, promote blood circulation, and cleanse the body. In the past decade, pharmacological research revealed its potential to be applied to infectious disease for its numerous pharmacological activities such as anti-inflammatory [5–7], antimicrobial [3, 8], antifungal [3, 8, 9], antivirus [10], and immunoenhancing [3]. Recent studies have further researched Rhubarb as a treatment for sepsis in humans and animal models. However, assessment of efficacy and mechanisms of Rhubarb in treating sepsis still lack systematic analysis. Herein, we report a systematic review of the use of Rhubarb in experimental sepsis in this paper. The objectives of this study were to

1. systematically review and assess the experimental evidence for Rhubarb administered before or after onset of sepsis in animal models;
2. determine the efficacy of Rhubarb in sepsis and explore the impact on the efficacy of defined characteristics;
3. analyze the possible antisepsis mechanisms of Rhubarb;
4. propose the development for the design of future experimental sepsis and ultimately for further clinical trials in human patients.

## 2. Methods

### 2.1. Search Strategy

We identified studies of Rhubarb in animal models of sepsis from PubMed, the Cochrane Central Register of Controlled Trials (CENTRAL) in The Cochrane Library, EMBASE, Chinese National Knowledge Infrastructure (CNKI), VIP Database, Wanfang Data, and Chinese Biomedical Literature Database (CBM) by using the terms “Rheum” OR “Rhubarb” OR “emodin” OR “rhein” OR “Dahuang” AND “sepsis” OR “septicemia” OR “septic shock” OR “endotoxic shock” OR “toxic shock” OR “blood stream infection” in English or in Chinese (the search terms used in PubMed are listed in the appendix). All searches were limited to studies on animals without language restrictions from the inception of each aforementioned database up to December 2014. Reference lists of all included articles and relevant reviews were also handsearched. Two authors (Yan-na Weng and Jing-xia Zheng) identified studies from databases independently. Disagreements were solved after discussion with a third party (Yun Han).

### 2.2. Eligibility

Studies have to be concerning specifically the effectiveness of Rhubarb (regardless of dosage, form, maturity, mode of administration, course of treatment, or storage time before use) for sepsis. To be specific, the inclusion criteria were preset as follows for preventing bias: (1) studies that compared Rhubarb as monotherapy or an adjuvant therapy for experimental sepsis were included and (2) effectiveness was compared with control group receiving vehicle, no treatment, positive drug therapy, or the same supporting treatment (such as fluid resuscitation and antibiotics) as treatment group. Exclusion criteria were prespecified as follows: (1) studies comparing Rhubarb as a monotherapy to another type of Chinese herbal medicine (CHM); (2) duplicate publications; and (3) not focusing on experimental sepsis. Two authors (Dong-ping Xie and Shu-tao Mai) screened for included studies independently. Disagreements were solved after discussion with a third party (Yun Han).

### 2.3. Data Extraction

We extracted the following details from each study: (1) the first author's name and publication year; (2) model of sepsis, details of model induction method, anesthetized method, number of animals, animal species, sex, age, weight, comorbidities (hypertension, diabetes, aged, etc.), and study design; (3) type of Rhubarb treatment, dosage form, initial treatment performing time, course of treatment, dosage, administration mode, intervention of control group, and supportive care for animals; and (4) outcome measure, outcomes assessments time, and intergroup differences.

If the data were missing or conflicting, we tried to get further information by contacting the authors. The study would be excluded if no further information is available and the key data remain conflicting. Two authors (Yan Zhang and Jiong-dong Du) extracted data independently. Disagreements were solved after discussion with a third party (Yun Han).

### 2.4. Quality Assessment

We applied a modified scale to evaluate the methodological quality of the included studies [11–14]: (1) peer-reviewed publication; (2) control of temperature, humidity, and light; (3) randomized allocation; (4) reporting details of randomized allocation method; (5) allocation concealment; (6) blinded model induction; (7) blinded intervention administration; (8) blinded outcome assessment; (9) sample size calculation or explanation; (10) animal welfare regulations compliance statement; (11) being free of selective reporting; (12) potential conflict of interests statement; (13) reporting statistical method; (14) reporting numerical data in Result sections; (15) reporting actual numbers of animal samples of different groups in Result sections; (16) completeness of follow-up; (17) intention-to-treat analysis. Denotation was by a “+” when it was a “yes” for the item (attributed one point), a “±” when it was a “partially yes” (attributed 0.5 points), a “?” when it was “unclear” (attributed 0 points), and a “−” when it was a “no” (attributed 0 points). Two authors (Guang-ping Wu and Fang Lai) assessed study quality independently. Discussions were carried out with a third party (Yun Han) to solve any disagreements.

## 3. Results

### 3.1. Study Selection

**Figure 1:**
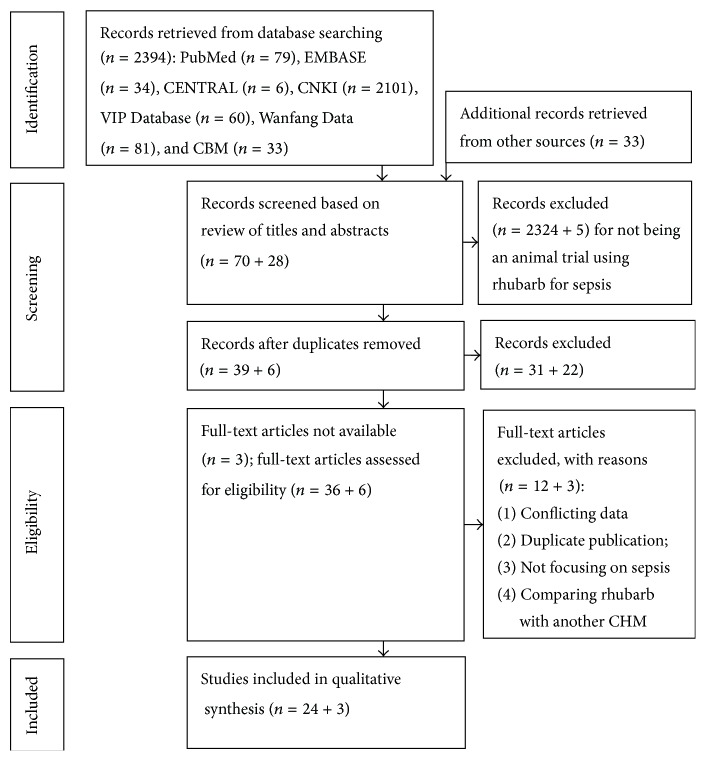
Flow diagram.

2394 papers were retrieved from seven databases. 70 articles remained after screening by titles and abstracts, of which 31 records were removed for being duplicates. Another 3 articles were excluded for no full text was available with effort. By full-text reviewing, 12 studies were excluded according to the exclusion criteria. Three more eligible studies were included after handsearching of reference lists of the included articles and other relevant reviews. Finally, a total of 27 studies were included in qualitative synthesis [15–41]. The study selection process is summarized in a flow diagram (Figure 1).

### 3.2. Characteristics of Included Studies

#### 3.2.1. Time and Place of Studies

The 27 included trials were published or written (if it was an unpublished thesis) between 1992 and 2014. 14 of the 27 trials were within the last 5 years. All were conducted in China.

#### 3.2.2. Experimental Animals

The animal species included Wistar rats, Sprague-Dawley (SD) rats, Kunming mice, ICR mice, piglets, and New Zealand rabbits. 77.8% of the trials utilized male animals (21 out of the 27 trials), while only 2 trials [23, 35] used both male and female animals and 4 studies [15, 17–19] did not report the sex of animals. Only one study [26] reported the specific age of animals, 3 studies [35, 40, 41] reported using adult animals, and the rest 23 studies had no statement about animals' age. 10 of the included articles reported that all animals utilized were healthy, 2 articles [28, 31] utilized specific pathogen-free (SPF) animals, and another 2 articles [33, 37] reported using “clean animals” (as described in the original text), while 13 articles did not report whether there were any comorbidities in animals.

#### 3.2.3. Sepsis Models

11 of the 27 studies (40.7%) were lipopolysaccharide (LPS) injection model, the dosage of LPS varied from 4 mg/kg to 15 mg/kg in rodents and 0.01 mg/kg for piglets, 6 studies [16, 30, 31, 33, 37, 39] administrated LPS through intraperitoneal (IP) injection, and 5 studies [18, 20, 21, 26, 34] administrated through intravenous (IV) injection. 29.6% (8 out of 27) [23, 25, 27, 28, 35, 36, 40, 41] of the included studies utilized cecal ligation and puncture (CLP) model. The length of the ligated cecum varied from 1/3 to the whole length of cecum. Most of the studies reported one-time puncture (3 of the 8 reports) [36, 40, 41], using 18-gauge needles for puncture (6 of the 8 reports) [23, 27, 28, 35, 36, 40] and did not report the type of suture used for ligation (5 out of 8 studies) [23, 27, 28, 36, 41]. Moreover, there were some studies not reporting the length of the ligated cecum [23] or times of perforation administration [23, 27]. Pentobarbital [25, 27, 29, 32, 36] and urethane [18, 20, 34, 40, 41] were used in 5 studies to induce anesthesia, respectively, ketamine [22–24, 38] in 4 studies, chloral hydrate [35, 37, 39] in 3 studies, and xylazine hydrochloride [28] in 1 study, and the remaining 9 studies had no statement about anesthetics.

The model details of included studies are summarized in Table 1.

#### 3.2.4. Sample Sizes

The design number of animal in TG (treatment group) for each outcome index at each time phase varied from 5 to 15, while 2 articles [17, 31] did not report the design number of animals in each subgroup. 10 articles set the animals number in TG at 8, 6 articles set at 6, 4 articles set at 5, 3 articles set at 10, and the rest 2 articles set at 14 and 15, respectively. 10 articles reported the actual numbers of animal samples of different groups in Result sections; 8 articles partially reported, while 9 articles had no report on numbers of samples in Result sections.

#### 3.2.5. Rhubarb Intervention

Six studies [27, 34, 37, 39–41] used emodin, one of the active ingredients in Rhubarb, as the intervention for TG. The dosage varied from 20 mg/kg to 60 mg/kg per time and from 20 mg/kg to 300 mg/kg in total during course of treatment. Three studies [27, 40, 41] were intragastric (IG) administration, 2 studies [37, 39] were IP, and 1 study [34] was enema. Only one study [33] used rhein, another kind of active ingredients in Rhubarb, as treatment for TG. The rest 20 studies all reported using rhubarb in TG; however, none of these studies tested the actual active ingredients of the Rhubarb therapy they used. Three articles [15, 28, 32] reported using raw Rhubarb, while the remaining 17 articles did not mention the maturity of Rhubarb. Powder [20, 22, 24, 28, 32, 35] form of Rhubarb was used in 6 studies, decoction [15, 25, 31] and extract (no available details) [17, 21, 23] of Rhubarb were used in 3 studies, respectively, granule [30, 36] was used in 2 studies, and the rest 6 studies had no statement about the dosage form of Rhubarb therapy. Most of the studies (85%, 17 out of 20) administrated Rhubarb therapy by IG injection; only one study [21] was by IP injection and the remaining 2 studies [17, 26] did not mention administration method of Rhubarb therapy.

#### 3.2.6. Supportive Therapies

Eight studies offered description of supportive therapy in the articles. Among them, fluid resuscitation therapy [24, 28, 32, 35, 36, 38] was utilized in 6 studies, keeping warm [25, 35] in 2 studies, and antibiotics [29] in 1 study. Balanced salt solution (BSS), Ringer Solution (RS), and normal saline (NS) were used for fluid resuscitation; the dosage varied from 30 mL/kg to 100 mL/kg, administrated by IV, IP, or hypodermic injection. The rest 19 studies had no statement about supportive therapy in the articles.

### 3.3. Quality of Studies and Publication Bias

The scores of study quality checklist (SQC) varied from 2 to 6.5 out of a total of 17 points, with the median score of 4. Only 5 out of the 27 articles [17, 25, 34–36] described control of temperature, humidity, and light. 21 articles claimed using randomized allocation; however, none of the articles reported specific details of randomized allocation method or allocation concealment. Only one study [31] reported using blinded model induction method. No study had statement about blinded intervention administration, blinded outcome assessment, sample size calculation or explanation, or being free of selective reporting. Only 2 articles [34, 38] made a potential conflict of interests statement. Two studies [15, 17] did not describe statistical method. One article [34] reported all the outcomes graphically, while 12 articles [16, 17, 21–26, 29, 33, 37, 41] reported partially in graphical way and partially in numerical way, and the rest 14 articles reported all the data numerically. Only one study [17] completed follow-up of animals' survival rates up to 40 days, and none of the 27 studies utilized intention-to-treat analysis when dealing with data of animals that died long before samples could be taken according to schedule. The checklist for study quality and risk of bias are shown in Table 2.

### 3.4. Effectiveness

Meta-analysis could not be carried out due to the high heterogeneity and low methodological quality of the studies.

#### 3.4.1. Effects of Rhubarb on Gastrointestinal Biological Barrier in Experimental Sepsis

Three studies [29, 31, 32] reported significant effects of Rhubarb on restoring balance of gastrointestinal microflora during sepsis, manifesting in less loss of normal gastrointestinal flora (such as* Bifidobacterium* [31] and normal colibacillus [29]) and less growth of opportunistic enteropathogenic bacteria (such as* Escherichia coli*) [31, 32].

#### 3.4.2. Effects of Rhubarb on Gastrointestinal Mechanical Barrier in Experimental Sepsis

Rhubarb therapies were reported significantly reducing injuries of gastrointestinal mucosa [16, 18, 19, 26, 27, 35], decreasing intestinal microvascular permeability [18, 19, 26, 27], improving microcirculation [27, 34, 36], and ameliorating metabolism [36] of intestine during sepsis. Resistance to apoptosis of intestinal epithelial cells in sepsis animals treated with Rhubarb was also described [26].

Lower incidence of bacterial translocation to remote organs [16, 29] during severe sepsis might be a result of the effects of Rhubarb on gastrointestinal biological and mechanical barrier.

#### 3.4.3. Effects of Rhubarb on Lung Microvascular Permeability in Experimental Sepsis

Five studies [21, 25, 37, 39, 40] described results of significant lower wet to dry ratio (W/D) of lung tissue when sepsis animals were treated with Rhubarb therapies, as well as less bronchoalveolar lavage fluid (BALF) neutrophils count [21, 23, 25] and BALF protein content [21, 25], indicating that Rhubarb might be effective in inhibiting leakage of leukocyte and protein from vessels to lung tissue [21, 23, 25, 40] during sepsis and finally reducing sepsis-induced lung injury [21, 23, 25, 37, 41].

#### 3.4.4. Effects of Rhubarb on Liver Injury in Experimental Sepsis

Chen [24] reported that Rhubarb therapy could protect liver from injury during sepsis. The effects of reducing the loss of liver mitochondria cytochrome C ATP and liver mitochondria cytochrome oxidase, and inhibiting inflammation by suppressing mRNA expression of inflammatory cytokines (TNF-*α* and IL-1*β*) and their receptors, might be the mechanism of the protective effect.

#### 3.4.5. Potential Mechanisms of the Protective Effects of Rhubarb in Experimental Sepsis

It was described in studies that Rhubarb therapies could suppress production of lipid peroxides (LPO) [16], reduce content and activity of malondialdehyde (MDA) [25, 26, 33, 39], inhibit activity of myeloperoxidase (MPO) [25, 34], increase superoxide dismutase (SOD) [16, 24–26, 33, 39] activity, and upregulate catalase (CAT) and glutathione-peroxidase (GSH-Px) [39] activity in sepsis animals, indicating that Rhubarb might be able to reduce oxidative stress during sepsis. Also, Rhubarb therapies were reported as being capable of suppressing gene expression of TNF-*α* [24, 26], IL-1*β* [24], and IL-10 [26], downregulating expression of tumor necrosis factor receptors (TNFR) [22, 24], decreasing endotoxin levels [15, 23, 24, 33], suppressing activity of phospholipase A_2_ (PLA_2_) [20], reducing production of platelet activating factor (PAF) [20], and decreasing secretion of IL-6 [24, 39], IL-8 [30], IL-17 [39], high mobility group protein box-1 (HMGB1) [28], TNF-*α* [22–24, 27, 28, 30, 33, 35, 37], IL-1*β* [33, 37], and IL-10 [33], demonstrating Rhubarb's potential of reducing inflammation in sepsis. Li et al. reported that emodin significantly inhibited degranulation of mast cells induced by LPS and reduced expression of L-selectin, intercellular adhesion molecule 1 (ICAM-1), CD11b, activator protein-1 (AP-1), toll-like receptor 4 (TLR4), and nuclear factor kappa B p65 (NF-*κ*B p65), suggesting emodin's beneficial role in ameliorating microcirculatory disturbance (adhesion molecule expression, leukocyte adhesion, and cytokine release) by a possible pathway involving TLR4, NF-*κ*B, and AP-1 in sepsis animals [34]. Zhang et al. described a protective effect of Rhubarb on maintaining immune balance in sepsis rats by upregulating CD4+ lymphocytes expression and restoring an approximately normal level of CD4+/CD8+ ratio [38]. Sun et al. reported that lung AQP-1 mRNA and protein expression were significantly suppressed by emodin, suggesting that it might be one of the mechanisms of effect against pulmonary edema [41].

The detailed outcome indexes of included studies are summarized in Table 3.

## 4. Discussion

The present study is the first systematic review of Rhubarb for animal model of sepsis in both English and Chinese literatures. The present study showed that Rhubarb might be effective in reducing injuries induced by sepsis in gastrointestinal tract, lung, and liver, and its potential mechanisms for antisepsis might include reducing oxidative stress and inflammation, ameliorating microcirculatory disturbance of adhesion molecule expression, leukocyte adhesion and cytokine release, and maintaining immune balance during sepsis.

However, there was significant heterogeneity among the included studies, which might affect the effectiveness of Rhubarb therapy. From analysis of the heterogeneity, we can conclude several implications for further research.

Firstly, heterogeneity of Rhubarb intervention treatment was one of the most important factors.

There are several active constituents known about Rhubarb, including anthraquinone derivatives, such as emodin, chrysophanol, rhein, physcion, and their glycoside compounds, and stilbene derivatives such as piceatannol, resveratrol, and their glycoside derivatives [3]. Besides, there are several isolated complex compounds (e.g., torachrysone-8-O-*β*-D-glucopyranoside, sulphated emodin glucoside, and piceatannol-4′-O-*β*-D-(6′′-O-p-coumaroyl)-glucopyranoside) [3]. Pharmacological research on Rhubarb indicates that different aforementioned constituents have distinct pharmacological characteristics. Chrysophanol-8-O-glucoside was reported to be the most effective inhibitor on platelet aggregation induced by collagen and thrombin comparing to the other three kinds of Rhubarb extract (chrysophanol, emodin, and physcion) [44]. The total extract of Rhubarb (extracted with 60% ethanol) and the total anthraquinones extract were reported to be purgative active, with the latter one being stronger than the former one, while successive dosage of total tannins extract from Rhubarb produced an antidiarrheal activity [45]. Furthermore, the contents of different constituents from Rhubarb vary when it is under different processed method (raw, wine-processed, vinegar-processed, etc.) [46] with different vehicles (water, ethanol, etc.) [47] in different dosage forms (granule, powder, slice, etc.) [47] and the pharmacological effects could be of great difference [48, 49]. Also, pharmacokinetics are different when it comes to different target organs [50], administration methods [51], and age groups [52]. Therefore, further study of Rhubarb or other CHM should pay attention to identification of exact contents of different constituents with a clear statement about the dosage form, process procedure, administration method, and so forth and draw more consideration about type and dosage of Rhubarb or its active ingredients according to different targets.

Secondly, heterogeneity of sepsis animal models was another very important factor.

The animal model for sepsis in the included studies mainly lies in LPS injection model (including LPS+ model like scald plus LPS injection) and CLP model. LPS model is notable for the advantages of simple, reproducible, highly controlled, and standardized administration, while, at the same time, the disadvantages of it cannot be easily overlooked: early, high, and transient increases of inflammatory mediators through a TLR4-dependent pathway, different hemodynamic response from human sepsis, not being able to reflect all complex physiological responses in human sepsis, and variability in dose and administration route [53–57]. The literature indicated that LPS administrated intraperitoneally induced systematic responses mainly via the lymphogenous route rather than the hematogenous route. Portal LPS can be effectively eliminated by the liver and does not reach the systemic circulation at all unless the LPS concentration is extremely high. Besides, intraperitoneal administration of LPS is transported mainly in lipoprotein-bound form, which is less active in inducing systematic responses [58]. It had been reported that the dose of intraperitoneally administered LPS was approximately 100 times of the intravenous dose in order to produce a similar peak rise in temperature in New Zealand White rabbits [59]. However, the effects of different LPS administration doses or routes on course or outcomes of sepsis still deserve further investigation.

Comparing to LPS model, CLP model is characterized by its similar hemodynamic, immune, and metabolic phases to human sepsis by recreating human polymicrobial sepsis progression. The CLP model has even been titled with “the golden model” for sepsis [55, 57]. Yet the complexity of human sepsis still cannot be completely reproduced by CLP model and the severity varies by differences in model procedures [55–57], including needle size for puncture, number of cecal punctures, type of suture, operation incisions, and volume of cecal contents extruded [57, 60].

Pentobarbital, urethane, chloral hydrate, and ketamine are commonly used anesthetic drugs in animal experiment research. Studies demonstrated that these anesthetics had significantly different effects on systemic hemodynamics [61, 62], organ functions, and immune response [63, 64] in experimental animals. It was reported that chloral hydrate (300 and 400 mg/kg) severely depressed the cardiovascular and respiratory systems in rats, while pentobarbital (40 mg/kg) produced a moderate to severe depression and urethane (1.2 and 1.5 g/kg) was at a moderate level [65]. Urethane induced apoptosis in kidney, while pentobarbital had little effect on renal cell apoptosis [66]. Although pentobarbital augmented LPS-induced hypotension, it also attenuated LPS-induced acute lung injury (ALI) and organ dysfunctions [67]. Ketamine inhibited hypotension, suppressed cytokine responses, and reduced organ dysfunction in sepsis animals in vivo [68, 69], but its depression on cardiac function of isolated rat hearts was significantly greater than pentobarbital and chloral hydrate [70]. Reevaluation should be taken into consideration when interpreting the results of experiments anesthetized with these anesthetic agents. Besides, it is worth noting that, in experimental sepsis, there have been no comprehensive comparisons among all these anesthetics. Which anesthetic is the most suitable agent for sepsis experiments remains unknown.

Meanwhile, as the elderly population is the major population of sepsis and age is an independent predictor of mortality, aged animals should be under consideration for experimental sepsis [53, 56]. Likewise, comorbidities that alter cardiovascular and immune function should be involved [56]. Also, gender selection is another crucial element since estrogen was found to be protective in immune function during experimental sepsis [56]. Not to mention the environmental controls and medication (including supportive therapies) that might affect outcomes of sepsis animals should also be taken into consideration to mimic that of human sepsis [56].

Besides, neotype sepsis models has been invented to reproduce various sepsis physiological progressions [54, 71]. So further studies for experimental sepsis should take the characteristics of different sepsis animal model into consideration and choose the most appropriate model.

Thirdly, quality of experimental design and reporting should be optimized.

A thorough protocol published before performance of experiments could reduce the risk of selective reporting. Journals can offer experiment reporting guidelines and carry out more restrictive policies for articles publication to help improve reporting quality. Statement of animal welfare compliance and sample size calculation are not criteria for risk of bias in animal trials, but they are important characteristics for evaluating the quality of evidence [11]. It is reported that risk of observer bias exists when animal model experiments lack blinding of outcome assessors [72]; however, experimental studies can almost always be blinded from allocation to model induction, intervention administration, and outcome assessment. Randomization process should be well designed and in detail reported to provide a more reliable basis for translational medicine from “bench to bedside.” Numerical data, both in animal numbers (no matter in Method section or Result section) and outcome measure results, should be advocated as it is more valuable for further assessment or reanalysis of the results. Furthermore, reasonable design of animal group, completeness of follow-up, and objective process of incomplete data are crucial elements for a sound experiment design.

Moreover, there are several limitations in this systematic review. First, the literatures of languages other than Chinese and English were not included in this systematic review, to some extent that might result in selective bias. Second, most of the studies were published articles (24 out of 27 studies); all of the data were collected from the published papers without more information accessed from the authors after trying to contact with them. As a result, the efficacy of Rhubarb might be overestimated due to publication bias. Third, general methodological quality of included studies was poor, indicating that caution was needed when interpreting the results.

## 5. Conclusions

There is no similar systematic review on Rhubarb for sepsis. 27 studies were identified from 7 databases in this systematic review to evaluate the efficacy of Rhubarb therapy for sepsis. Rhubarb has been apparently reported to be effective in reducing injuries induced by sepsis. Yet the positive findings should be interpreted with caution due to poor methodological quality. Rhubarb might be a promising candidate that is worth further clinical and experimental trials for sepsis.

## Figures and Tables

**Table 1:** Model details of included trials.

Included trials (years)	Model details
Zhao et al. (1992) [15]	Pneumococcus, ID, 0.2 mL, 30 million of bacteria count
Chen et al. (1994) [16]	LPS 0.4 mg, IP
Luo et al. (1996) [17]	HSV right cerebrum injection, virus titer: 100 LD50/0.1 mL, 0.02 mL per mouse
Chen et al. (1997) [18]	LPS 4 mg/kg, IV
Yang et al. (1998) [19]	NA
Yang et al. (1998) [20]	LPS 4 mg/kg, IV
Li et al. (2000) [21]	LPS 5 mg/kg, IV
Li and Chen (2001) [22]	Scald: 30%, third degree; LPS: 20 mg/kg, 12 h after scald procedure
Shu et al. (2003) [23]	CLP, using 18-gauge needles
Chen (2003) [24]	Scald: boiling water for 12 s, 25%, third degree; LPS: 20 mg/kg dissolved in 10 mL NS, 1 mL/h, 12 h after scald procedure
Yin et al. (2003) [25]	CLP, ligated 2/3 cecum with 1-0 suture, perforated 6 times with 7-gauge needles
Zhang et al. (2007) [26]	LPS 10 ug/kg, IV
Wu et al. (2007) [27]	CLP, ligated 1/3 cecum, perforated with 18-gauge needles
Xing et al. (2009) [28]	CLP, ligated cecum at 1 cm to ileocecal junction, perforated twice with 18-gauge needles
Chen et al. (2009) [29]	Scald: boiling water for 15 s, 30%, third degree; LPS: 10 mg/kg, q12 h × 2 times, 24 h after scald procedure
Han et al. (2011) [30]	LPS 6 mg/kg, IP
Huang et al. (2012) [31]	LPS 15 mg/kg, IP
Ma et al. (2012) [32]	Scald: boiling water for 15 s, 30%, third degree; LPS: 10 mg/kg, bid × 2 times, 24 h after scald procedure
Ma et al. (2012) [33]	LPS 10 mg/kg, IP
Li et al. (2013) [34]	LPS 5 mg/kg/h IV for 90 mins
Li (2013) [35]	CLP, ligated cecum with number 4 suture at 1.5 cm to ileocecal junction, perforated 3 times with 18-gauge needles at 1 cm to ileocecal junction
Cui (2013) [36]	CLP, ligated 50% of the cecum, perforated once with 18-gauge needles
Chen et al. (2013) [37]	LPS 10 mg/kg, IP
Zhang et al. (2014) [38]	Scald: boiling water for 12 s, 30%, third degree; LPS: 5 mg/kg, 12 h after scald procedure
Su (2014) [39]	LPS 10 mg/kg, IP
Liu et al. (2014) [40]	CLP, ligated at ileocecal junction with 4-0 suture, perforated once with 18-gauge needles
Sun et al. (2014) [41]	CLP, ligated at ileocecal junction, perforated once with 21-gauge needles

*Note*. HSV: herpes simplex virus; NA: not available.

**Table 2:** Study quality and risk of bias.

Study	(1)	(2)	(3)	(4)	(5)	(6)	(7)	(8)	(9)	(10)	(11)	(12)	(13)	(14)	(15)	(16)	(17)	Total				Scores
																		+	±	?	−	
Zhao et al. (1992) [15]	+	−	−	−	−	−	−	−	−	−	−	−	−	+	+	−	−	3	0	0	14	3
Chen et al. (1994) [16]	−	−	−	−	−	−	−	−	−	−	−	−	+	±	±	−	−	1	2	0	14	2
Luo et al. (1996) [17]	?	+	−	−	−	−	−	−	−	−	−	−	−	±	−	+	−	2	1	1	13	2.5
Chen et al. (1997) [18]	+	−	−	−	−	−	−	−	−	−	−	−	+	+	−	−	−	3	0	0	14	3
Yang et al. (1998) [19]	−	−	+	−	−	−	−	−	−	−	−	−	+	+	−	−	−	3	0	0	14	3
Yang et al. (1998) [20]	+	−	+	−	−	−	−	−	−	−	−	−	+	+	+	−	−	5	0	0	12	5
Li et al. (2000) [21]	+	−	−	−	−	−	−	−	−	−	−	−	+	±	±	−	−	2	2	0	13	3
Li and Chen (2001) [22]	?	−	+	−	−	−	−	−	−	−	−	−	+	±	±	−	−	2	2	1	12	3
Shu et al. (2003) [23]	?	−	+	−	−	−	−	−	−	−	−	−	+	±	−	−	−	2	1	1	13	2.5
Chen (2003) [24]	/^☆^	−	+	−	−	−	−	−	−	−	−	−	+	±	±	−	−	2	2	0	12	3
Yin et al. (2003) [25]	?	+	+	−	−	−	−	−	−	−	−	−	+	±	−	−	−	3	1	1	12	3.5
Zhang et al. (2007) [26]	+	−	+	±^⋄^	−	−	−	−	−	−	−	−	+	±	±	−	−	3	3	0	11	4.5
Wu et al. (2007) [27]	+	−	+	−	−	−	−	−	−	−	−	−	+	+	+	−	−	5	0	0	12	5
Xing et al. (2009) [28]	?	−	+	−	−	−	−	−	−	−	−	−	+	+	−	−	−	3	0	1	13	3
Chen et al. (2009) [29]	+	−	+	±^◆^	−	−	−	−	−	−	−	−	+	±	±	−	−	3	3	0	11	4.5
Han et al. (2011) [30]	+	−	+	±^◆^	−	−	−	−	−	−	−	−	+	+	+	−	−	5	1	0	11	5.5
Huang et al. (2012) [31]	+	−	+	±	−	+	−	−	−	−	−	−	+	+	+	−	−	6	1	0	10	6.5
Ma et al. (2012) [32]	+	−	+	−	−	−	−	−	−	−	−	−	+	+	+	−	−	5	0	0	12	5
Ma et al. (2012) [33]	+	−	+	±^★^	−	−	−	−	−	−	−	−	+	±	±	−	−	3	3	0	11	4.5
Li et al. (2013) [34]	+	+	+	−	−	−	−	−	−	+	−	−	+	−	−	−	−	5	0	0	12	5
Li (2013) [35]	/^☆^	+	+	±^◆^	−	−	−	−	−	−	−	−	+	+	+	−	−	5	1	0	10	5.5
Cui (2013) [36]	/^☆^	+	+	−	−	−	−	−	−	−	−	−	+	+	−	−	−	4	0	0	12	4
Chen et al. (2013) [37]	+	−	+	−	−	−	−	−	−	−	−	−	+	±	−	−	−	3	1	0	13	3.5
Zhang et al. (2014) [38]	+	−	+	±^◆^	−	−	−	−	−	+	−	−	+	+	+	−	−	6	1	0	10	6.5
Su (2014) [39]	+	−	+	±^◆^	−	−	−	−	−	−	−	−	+	+	+	−	−	5	1	0	11	5.5
Liu et al. (2014) [40]	−	−	+	−	−	−	−	−	−	−	−	−	+	+	+	−	−	4	0	0	13	4
Sun et al. (2014) [41]	+	−	−	−	−	−	−	−	−	−	−	−	+	±	±	−	−	2	2	0	14	3

*Note*. (1) Peer-reviewed publication; (2) control of temperature, humidity, and light; (3) randomized allocation; (4) reporting details of randomized allocation method; (5) allocation concealment; (6) blinded model induction; (7) blinded intervention administration; (8) blinded outcome assessment; (9) sample size calculation or explanation; (10) animal welfare regulations compliance statement; (11) being free of selective reporting; (12) potential conflict of interests statement; (13) reporting statistical method; (14) reporting numerical data in Result sections; (15) reporting actual numbers of animal samples of different groups in Result sections; (16) completeness of follow-up; (17) intention-to-treat analysis. **+**: yes, scores 1 point; **−**: no, scores 0 points; ±: partially yes, scores 0.5 points; ?: unclear, scores 0 points; ^☆^unpublished doctor's or master's thesis; ^⋄^randomized allocation according to body weight; details are unavailable; ^◆^randomized allocation according to random number table; details are unavailable; ^★^completely randomized design; details are unavailable.

**Table 3:** Outcome index of included trials.

Included trials (years)	Outcome indexes (time), intergroup differences^*▽*^
Zhao et al. (1992) [15]	ETX in blood (q12 h from 0 h to 48 h), *P* < 0.01

Chen et al. (1994) [16]	(1) Bacteria culture of swabs of liver, spleen, and mesentery (24 h) and LPO level of liver and small intestine (24 h), *P* < 0.01 (2) SOD content of liver, small intestine, and plasma (24 h), *P* < 0.05

Luo et al. (1996) [17]	(1) Survival rates (40 d); TG survival rates were higher, *P* value NA (2) HSV antigen in brain, heart, liver, and ganglion (the 2nd, 4th, 6th, 8th, 10th, 12th, 16th, 20th, 30th, and 40th days); TG HSV antigen titer was lower, *P* value NA

Chen et al. (1997) [18]	Intestine *W*/*D* ratio and IVP (4 h AMI), *P* < 0.05

Yang et al. (1998) [19]	MAP, *W*/*D* of small intestine and ITPD (4 h AMI), *P* < 0.01

Yang et al. (1998) [20]	(1) MAP, plasma, and intestinal PLA_2_ level, intestinal PAF level (4 h AMI), *P* < 0.01 (2) Plasma PAF level (4 h AMI), *P* < 0.05

Li et al. (2000) [21]	(1) Lung *W*/*D* ratio (2 h AMI), *P* < 0.01 (2) BALF neutrophil rate and protein content, LPI and PVP, plasma NO, and lung iNOs level (2 h AMI), *P* < 0.05

Li and Chen (2001) [22]	(1) Plasma TNF-*α* level (24 h ASP), *P* < 0.001 (2) TNFR1 and TNFR2 DNA expression of small intestine (24 h ASP), lower TNFR1 and TNFR2 expression in TG, *P* value NA

Shu et al. (2003) [23]	(1) PVP and lung endotoxin level (0 h, 24 h, 48 h, 72 h, 96 h, and 120 h), *P* < 0.05 (48 h, 72 h, 96 h, and 120 h) (2) Lung TNF-*α* level (0 h, 24 h, 48 h, 72 h, 96 h, and 120 h), *P* < 0.01 (48 h, 72 h, 96 h, and 120 h) (3) Neutrophil percentage in BALF (0 h, 24 h, 48 h, 72 h, 96 h, and 120 h), lower percentages of neutrophils and macrophages in TG (48 h, 72 h, 96 h, and 120 h), *P* < 0.05

Chen (2003) [24]	(1) Liver TNF-*α* gene expression, TNF-*α* levels in plasma and liver and plasma IL-6 level (24 h ASP), *P* < 0.001 (2) Liver IL-1*β* gene expression (24 h ASP), no IL-1*β* gene expression in TG (3) Plasma endotoxin level (24 h ASP), *P* < 0.01 (4) TNFR1 and TNFR2 DNA expression of liver (24 h ASP), lower TNFR1 and TNFR2 expression in TG, *P* value NA (5) Liver mitochondria cytochrome C ATP and EC levels (2 h, 4 h, and 8 h ALPSI), *P* < 0.001 (6) Liver mitochondria cytochrome oxidase level and SOD levels in plasma, small intestine, and liver (2 h, 4 h, and 8 h ALPSI), *P* < 0.01

Yin et al. (2003) [25]	Lung *W*/*D*, BALF neutrophil percentage and BALF protein content; LPI and PVP; lung MDA, SOD, and MPO (the 16th day), *P* < 0.01

Zhang et al. (2007) [26]	IEC apoptosis; intestinal TNF-*α* mRNA expression, IL-10 mRNA expression, and ZO-1/occludin mRNA expression; intestinal MDA and SOD levels (NA), *P* < 0.01

Wu et al. (2007) [27]	Plasma TNF-*α* level, MV BFV, PLF protein/plasma protein level ratio, and pathological scores^*∗*^ of intestinal mucosa (72 h), *P* < 0.05

Xing et al. (2009) [28]	(1) Plasma TNF-*α* level (0 h, 3 h, 8 h, 24 h, 48 h, and 72 h), *P* < 0.05 (8 h) (2) Plasma HMGB1 level (0 h, 3 h, 8 h, 24 h, 48 h, and 72 h), *P* < 0.05 (24 h, 48 h, and 72 h)

Chen et al. (2009) [29]	(1) Gut enteric bacilli count (the 1st, 3rd, and 9th days), *P* < 0.01 (the 3rd and 9th days) (2) Bacteria species in gut (the 1st, 3rd, and 9th days), less colibacillus loss in TG, *P* value NA (3) Bacterial translocation (the 1st, 3rd, and 9th days), *P* < 0.05 (the 1st day)

Han et al. (2011) [30]	(1) Plasma TNF-*α* and IL-8 level (24 h, 48 h, and 72 h), *P* < 0.01 (2) Pathological scores^*∗*^ of intestinal mucosa (72 h), *P* < 0.01

Huang et al. (2012) [31]	(1) Gut *Enterococcus* count, lactobacilli count, and aerobe count (the 10th day), *P* > 0.05 (2) Gut *Bifidobacterium* count, *Escherichia coli* count, and anaerobe count; gut anaerobe to aerobe ratios (the 10th day), *P* < 0.05

Ma et al. (2012) [32]	(1) Gut enteric bacilli count (24 h), *P* < 0.01 (2) Gut lactobacilli count, colon content, gut monilia count, and species (24 h), *P* > 0.05

Ma et al. (2012) [33]	(1) Plasma endotoxin, TNF-*α* and IL-1*β* level; liver IL-10, MDA, and SOD level (8 h), *P* < 0.01

Li et al. (2013) [34]	(1) RBC velocity in MV (#), *P* < 0.05 (from 50 to 90 min ALPSI) (2) WSR (#), *P* > 0.05 (3) Leukocyte rolling velocity in MV (#), pretreatment: *P* < 0.05 (from 60 to 90 min ALPSI), posttreatment: *P* > 0.05 (4) Cell count of rolling leukocytes along MV walls (#), pretreatment: *P* < 0.05 (50 and 60 min ALPSI), posttreatment: *P* > 0.05 (5) Leukocyte adhesion to MV walls (#), pretreatment: *P* < 0.05 (from 20 to 90 min ALPSI), posttreatment: *P* > 0.05 (6) Leukocyte emigration out of MV; DHR fluorescence intensity ratio on MV walls; albumin leakage from MV (#), *P* < 0.05 (from 40 to 90 min ALPSI) (7) Mast cell degranulation, blood L-selectin, and CD11b (90 mins ALPSI), pretreatment: *P* < 0.05, posttreatment: *P* > 0.05 (8) Blood CD18 (90 min ALPSI), *P* > 0.05 (9) TLR4, NF-*κ*B p65, AP-1, MPO, and ICAM-1 expression in intestine (90 min ALPSI), *P* < 0.05

Li (2013) [35]	(1) Plasma DAO level (6 h, 12 h, 24 h, and 48 h), *P* < 0.01 (versus NTG, 24 h and 48 h), *P* > 0.05 (versus PCG) (2) Intestinal DAO level (48 h), *P* > 0.05 (3) Plasma TNF-*α* level (6 h, 12 h, 24 h, and 48 h), *P* < 0.01 (versus NTG, 6 h), *P* < 0.05 (versus NTG, 12 h), and *P* > 0.05 (versus PCG)

Cui (2013) [36]	(1) Intestinal wall blood flow, lactate/pyruvate ratio in jejuna, the area of intestinal mucosa capillary, and intestinal mucosa capillary count (24 h AMI), *P* < 0.01 (2) Intestinal mucosa blood flow (24 h AMI), *P* < 0.05

Chen et al. (2013) [37]	(1) Lung *W*/*D* ratio and plasma TNF-*α* and IL-1*β* level (6 h), *P* < 0.05 (2) Lung NF-*κ*B p65 (6 h), lower NF-*κ*B p65 expression in TG, *P* value NA

Zhang et al. (2014) [38]	(1) Peripheral blood leucocyte and hepatocyte GR binding capacity (12 h, 24 h, and 72 h ALPSI), *P* < 0.01 (2) CD4+ lymphocytes in peripheral blood (12 h, 24 h, and 72 h ALPSI), *P* < 0.01 (12 h and 24 h) (3) CD8+ lymphocytes in peripheral blood (12 h, 24 h, and 72 h ALPSI), *P* > 0.05 (4) CD4+/CD8+ ratio (12 h, 24 h, and 72 h ALPSI), *P* < 0.01 (24 h and 72 h)

Su (2014) [39]	(1) Plasma IL-6 and IL-17 level (12 h ALPSI), *P* < 0.05 (2) Lung *W*/*D* ratio; lung MDA, SOD, GSH-Px, and CAT level; lung pathological scores^*∗∗*^ (12 h ALPSI), *P* < 0.01

Liu et al. (2014) [40]	(1) Lung *W*/*D* ratio and LPI (3 h, 6 h, 12 h, and 24 h AMI), *P* < 0.05 (12 h and 24 h) (2) Lung pathological scores^*∗∗∗*^ (3 h, 6 h, 12 h, and 24 h AMI), *P* < 0.05 (24 h)

Sun et al. (2014) [41]	(1) Lung AQP-1 mRNA expression (3 h, 6 h, 12 h, and 24 h AMI), *P* < 0.05 (2) Lung AQP-1 protein expression (3 h, 6 h, 12 h, and 24 h AMI), *P* < 0.01 (6 h, 12 h, and 24 h AMI)

*Note*. ^*▽*^ *P* value of the comparison between treatment group and control group at each test time phase unless otherwise stated; ^*∗*^according to Chiu's method [42]; ^*∗∗*^according to Hofbauer's method [43]; ^*∗∗∗*^1 point for no injury, 2 points for 25% injuries, 3 points for 50%, 4 points for 75%, and 5 points for diffuse damage. #: every 10 min from 0 to 90 min ALPSI; ALPSI: after LPS injection; AMI: after model induction; AP-1: activator protein-1; AQP-1: aquaporin 1; ASP: after scald procedure; BFV: blood flow velocity; CAT: catalase; DAO: diamine oxidase; DHR: dihydrorhodamine; EC: energy charge; ETX: endotoxin concentration; GSH-Px: glutathione-peroxidase; GR: glucocorticoids receptor; ICAM: intercellular adhesion molecule; IEC: intestinal epithelial cells; ITPD: intestine transmural potential difference; IVP: intestinal vascular permeability, defined as the fluorescence protein content in intestinal tissue; TG: treatment group; LPI: lung permeability index, calculated by BALF protein to plasma protein ratio; LPO: lipid peroxides; MAP: mean arterial pressure; MDA: malondialdehyde; MPO: myeloperoxidase; MV: mesenteric venule; NA: not available; NTG: no treatment group; PAF: platelet activating factor; PCG: positive control group, the group animals that were treated with positive medicine; PLA_2_: phospholipase A_2_; PLF: peritoneal lavage fluid; PVP: pulmonary vascular permeability, defined as the Evans blue content in lung tissue; RBC: red blood cell; SOD: superoxide dismutase; TNF-*α*: tumor necrosis factor-*α*; *W*/*D*: wet to dry weight ratio; WSR: wall shear rate.

## Appendix

Search strategy for MEDLINE (PubMed)

#1 (Sepsis [Mesh]) OR (Septicemia) OR (Blood stream infection) OR (Septic Shock) OR (Endotoxic Shock) OR (Toxic Shock) OR (Severe sepsis)
#2 (Rheum [Mesh]) OR (Rhubarb) OR (emodin) OR (rhei) OR (rhein) OR (dahuang) OR (Da Huang) OR (Plants, Medicinal [Mesh]) OR (Pharmaceutical Plant) OR (Pharmaceutical Plants) OR (Medicinal Herb) OR (Medicinal Herbs) OR (Medicine, Chinese Traditional [Mesh])
#3 ((randomized controlled trial [pt]) OR (controlled clinical trial [pt]) OR (randomized [tiab]) OR (placebo [tiab]) OR (drug therapy [sh]) OR (randomly [tiab]) OR (trial [tiab]) OR (groups [tiab])) AND (animal [mh])
#4 #1 AND #2 AND #3.
